# Valorization of Bark from Short Rotation Trees by Temperature-Programmed Slow Pyrolysis

**DOI:** 10.1021/acsomega.1c00434

**Published:** 2021-03-31

**Authors:** Qing Zhao, Marko Mäkinen, Antti Haapala, Janne Jänis

**Affiliations:** † Department of Chemistry, University of Eastern Finland, Joensuu FI-80100, Finland; ‡ School of Forest Sciences, University of Eastern Finland, Joensuu FI-80100, Finland

## Abstract

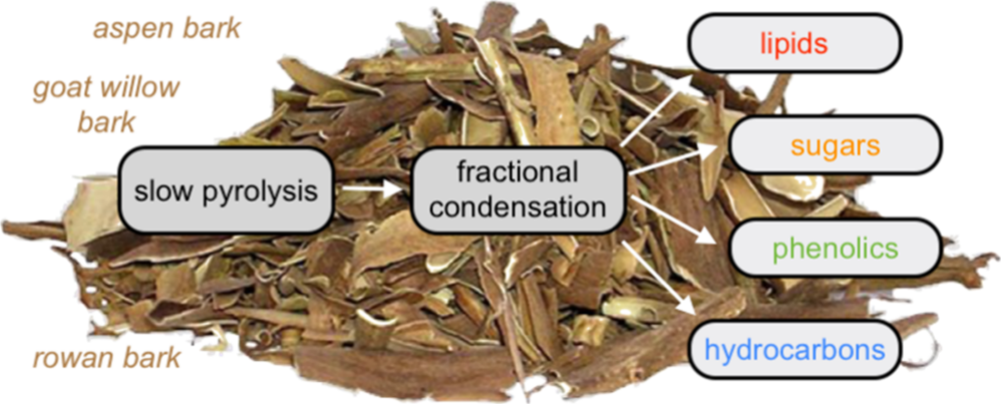

The tree bark represents an abundant but currently underutilized forest biomass side stream. In this work, temperature-programmed slow pyrolysis with fractional condensation was used for thermochemical conversion of the bark obtained from three short rotation tree species, aspen, goat willow, and rowan. Heating was performed in three stages, drying (135 °C), torrefaction (275 °C), and pyrolysis (350 °C), and the resulting vapors were condensed at 120, 70, and 5 °C, producing nine liquid fractions. An additional fraction was collected in the pyrolysis stage at 0 °C. The obtained liquid fractions were characterized in terms of their yields and bulk chemistry (i.e., CHNOS content, water content, pH, and total acid number) as well as their molecular level chemistry by high-resolution mass spectrometry. The highest liquid yields were obtained for the fractions condensed at 70 °C. The water content varied considerably, being the highest for the drying fractions (>96%) and the lowest for the pyrolysis fractions obtained at 120 °C (0.1–2%). Considerable compositional differences were observed between the liquid fractions. While the drying fractions contained mostly some dissolved phenolics, the torrefaction fractions contained more sugaric compounds. In contrast, the pyrolysis fractions were enriched lipids (e.g., suberinic fatty acids and their derivatives) and alicyclic/aromatic hydrocarbons. These fractions could be further refined into different platforms and/or specialty chemicals. Thus, slow pyrolysis with fractional condensation offers a potential route for the valorization of tree bark residues from forest industry.

## Introduction

Bark accounts typically for 9–15% of the dry weight of a tree log^1,2^ and is removed from the logs when the trees are processed in the saw or pulp mills. Some broad-leafed species have extremely high bark-to-wood ratios with a significant tree- and stand-level variation being reported.^3−5^ Tree bark represents the most abundant solid residue obtained from the forest industry that lacks valorization pathways to higher value-added products, beyond direct energy and process steam production via incineration or an environmentally questionable and costly landfilling. The chemical makeup of tree bark differs considerably from that of the stem wood; bark usually contains 2–6 times more lipophilic and hydrophilic extractives, higher content of lignin, and lower content of cellulose and hemicellulose. Wood extractives, in particular, possess a considerable utilization potential.^6^ Recent developments in the field also suggest that tree bark could be an interesting source of valuable phenolic compounds via thermal conversion or hydrolytic pathways.^7−15^

The more recently discovered end uses for industrial bark residues represent a wide array of structural, chemical, health-, and nutrition-related opportunities to complement the currently dominating energy use. For example, cork from the cork oak (*Quercus suber*) has been applied in construction, cosmetics, and pharmaceutical applications.^16^ Refined pine bark has been shown to function in bioherbicides and insecticides^17^ and in the recovery of metals from wastewater.^18^ Magnolia tree bark has shown anti-cancer, anti-inflammatory, anti-oxidant, and anti-depression activities,^19^ while valorization of different triterpenoids (e.g., betulin) and suberin from birch bark has also been extensively studied.^10,20−22^

These assessments have focused largely on the tree species of large-scale industrial uses in mechanical or chemical wood refining from which significant volumes of bark are generated as side streams. Far less attention has been given to the species that represent minor or no significant economic potential outside short rotation energy plantations due to their high carbon content and calorific value.^23^ In fact, the possibility to utilize biomass from short rotation coppice for chemical products is rather novel.^24,25^ The composition and concentrations of lignin and extractives differ markedly between species, stands, temporal zones, cambial age, and also between the inner and outer bark of the same trees.^26−29^ More detailed investigations on the bark fractions and their conversion pathways are still required to find their potential valorization opportunities.

Aspen, goat willow, and rowan are the examples of short rotation trees.^30−32^ Their bark has been shown to have a versatile chemical makeup,^31,33−36^ allowing extraction and purification of individual compounds, for example, for medical uses.^34,37^ New valorization routes for these unconventional yet quite abundant feedstocks would open new financial incentives and opportunities to increase the amount of deciduous broadleaf forests that is shown to positively affect the carbon cycle, surface energy fluxes, and ecosystem function, thereby modifying important feedbacks with the climate system.^38^

The extraction efficiency of individual chemical constituents greatly varies. The most common means to liberate these compounds from bark are hot water or solvent extraction.^39^ Pyrolysis, in comparison, is a cost-effective thermochemical method that possesses a great potential for large-scale conversion of tree bark residues^40^ but requires feedstock drying and yields a complex mixture of chemical degradation products. This can be mitigated to some extent by fractionation techniques that allow pyrolysis liquids to be separated into different compound classes. The major components in pyrolysis liquids are usually classified into carboxylic acids, esters, aldehydes, ketones, phenolics, aromatics, and organic nitrogenates with various ranges of percentages.^41^ A fractional condensation in conjunction with slow pyrolysis separates pyrolysis liquids directly into distinct compound fractions on the basis of their boiling points. As a result, the chemical properties of these fractions can better meet the specific post-processing requirements than the bulk liquids as a whole.^42−45^

Due to their complex chemical composition, pyrolysis liquids are challenging to characterize in detail.^43^ Conventional gas chromatography–mass spectrometry analyses are limited to the most volatile and low molecular weight constituents only and spectroscopic techniques give information mainly at the functional group level. High-resolution mass spectrometry has proven to be a powerful analytical tool for direct chemical fingerprinting of pyrolysis products, giving access to thousands of compounds, including the heavier and least volatile ones (e.g., anhydrosugars, polyphenolic constituents, and hydrocarbons).^10,43,46−49^ By using different ionization techniques, such as electrospray ionization (ESI) and atmospheric pressure photoionization (APPI), both polar and non-polar constituents present in pyrolysis liquids can be detected.

In this study, temperature-programmed slow pyrolysis with fractional condensation (Figure S1) was used for thermochemical conversion of bark from three short rotation tree species, namely, aspen (*Populus tremula*), goat willow (*Salix caprea*), and rowan (*Sorbus aucuparia*). Heating was performed in three stages (drying, torrefaction, and pyrolysis), and three distinct condensation temperatures were used at each stage, resulting in nine liquid fractions. An additional fraction was collected at 0 °C in the pyrolysis stage. The obtained liquid fractions were characterized with respect to their yields, bulk chemistry [CHNOS content, moisture, pH, and total acid number (TAN)], as well as their molecular level composition by high-resolution Fourier transform ion cyclotron resonance (FT-ICR) mass spectrometry (MS) to obtain an in-depth understanding on the chemical nature and valorization potential of these underused renewable resources.

## Results and Discussion

### Liquid Yields, Acidity, and Bulk Chemistry

The yields of the liquid fractions obtained and the bulk analysis results, including water content, pH, TAN, and elemental compositions, are shown in Table 1. The highest liquid yields were observed in the drying phase when the condensation temperature was 70 °C. On the other hand, these fractions had high water content (>96%), partly explaining the high liquid yields. Overall, all drying fractions, D-120, D-70, and D-5, had a very high water content as expected; at 135 °C, neither cellulose nor lignin is efficiently decomposed, although some extractives may be liberated and partial hemicellulose degradation may occur. Some liquid fractions, such as T-120 and P-70 for aspen and rowan and P-70 for goat willow, were phase-separated into aqueous and oily phases (AP and OP, respectively), increasing the total amount of the analyzed liquid fractions up to 12. The phase separation quite often occurs in the case of slow pyrolysis liquids due to their high water content.^47^ In our previous work with birch bark as feedstock, no phase separation was observed, however.^10^ The lowest water content was observed in the fractions P-120 and P-70/OP and in the case of rowan in P-0. The char yields were 32–39%, which are rather typical for slow pyrolysis oils.

**Table 1:** Yield, Water Content, pH, TAN, and Elemental Composition of Slow Pyrolysis Liquid Fractions

fraction^a^	yield^b^ (wt %)	water content (wt %)	pH	TAN (mg/g)	elemental composition^c^			
					C (wt %)	H (wt %)	N (wt %)	O (wt %)
Aspen (*Populus tremula*)								
D-120	2.3	98.4 ± 0.7	3.2	4.4 ± 0.1				
D-70	**13.6**	99.7 ± 0.3	3.2	2.4 ± 0.1				
D-5	6.2	96.5 ± 0.5	3.6	1.7 ± 0.1	58.8 ± 0.0	7.3 ± 0.1	19.7 ± 0.1	14.2 ± 0.2
T-120/OP	2.4	11.9 ± 0.3	N.A.^d^	74.2 ± 0.8	69.1 ± 0.0	8.0 ± 0.0	2.4 ± 0.0	20.5 ± 0.0
T-120/AP		88.0 ± 0.3	3.2	68.7± 0.1	63.3 ± 0.0	5.6 ± 0.0	4.8 ± 0.0	26.3 ± 0.0
T-70	**18.1**	94.0 ± 0.4	2.8	35.5 ± 0.1				
T-5	6.9	88.2 ± 0.3	2.9	32.2 ± 0.3	37.3 ± 0.0	7.8 ± 0.0	4.3 ± 0.1	50.6 ± 0.1
P-120	1.1	0.1 ± 0.1	4.5	61.9 ± 0.5	70.6 ± 0.2	8.5 ± 0.0	2.6 ± 0.0	18.3 ± 0.2
P-70/OP	1.4	6.3 ± 0.9	N.A.^d^	62.9 ± 0.5	74.3 ± 1.4	9.2 ± 0.0	2.1 ± 0.2	14.4 ± 1.3
P-70/AP		77.5 ± 0.7	4.1	86.2± 0.2	47.8 ± 0.1	7.0 ± 0.1	4.3 ± 0.0	40.8 ± 0.2
P-5	1.5	85.3 ± 0.8	3.3	57.1 ± 0.2	80.3 ± 0.9	6.2 ± 0.2	3.7 ± 0.1	9.8 ± 0.7
P-0	**2.1**	5.5 ± 0.9	4.0	47.2 ± 0.8	75.7 ± 1.2	9.4 ± 0.2	1.8 ± 0.7	13.1 ± 2.1
Goat willow (*Salix caprea*)								
D-120	0.1	99.9 ± 0.3	3.3	1.7 ± 0.1				
D-70	**8.1**	99.7 ± 0.7	2.9	2.8 ± 0.3				
D-5	7.4	99.6 ± 0.5	3.5	1.6 ± 0.1				
T-120	2	98.5 ± 0.1	2.9	19.1 ± 0.6				
T-70	**20.7**	95.2 ± 0.6	2.6	51.5 ± 0.2	67.8 ± 0.0	4.7 ± 0.0	14.9 ± 0.1	12.5 ± 0.2
T-5	6.8	93.4 ± 0.9	2.7	16.0 ± 0.6	32.8 ± 0.0	9.2 ± 0.0	7.4 ± 0.0	50.7 ± 0.0
P-120	0.4	2.0 ± 0.3	4.1	74.4 ± 0.7	65.5 ± 0.2	8.9 ± 0.1	2.5 ± 0.1	23.1 ± 0.3
P-70/OP	2.4	6.3 ± 0.8	N.A.^d^	96.4 ± 0.1	69.0 ± 0.9	9.0 ± 0.0	1.6 ± 0.0	20.4 ± 0.9
P-70/AP		75.6 ± 0.7	3.3	146.8 ± 0.7	51.0 ± 0.1	6.9 ± 0.2	2.8 ± 0.0	39.3 ± 0.3
P-5	**3.2**	83.0 ± 0.8	2.8	83.8 ± 0.0	40.3 ± 0.1	8.7 ± 0.1	2.4 ± 0.0	48.6 ± 0.1
P-0	0.9	90.0 ± 0.8	3.0	36.5 ± 0.4	40.4 ± 0.1	7.5 ± 0.0	3.4 ± 0.2	48.7 ± 0.2
Rowan (*Sorbus aucuparia*)								
D-120	0.1	99.1 ± 0.3	3.7	3.8 ± 0.2				
D-70	**13.4**	98.5 ± 0.5	3.6	3.1 ± 0.3	49.6 ± 0.1	8.9 ± 0.1	20.4 ± 0.1	21.2 ± 0.2
D-5	6.9	97.2 ± 0.5	3.6	2.3 ± 0.0	49.8 ± 0.0	9.4 ± 0.1	13.8 ± 0.1	27.0 ± 0.2
T-120/OP	0.3	33.3 ± 0.6	N.A.^d^	58.8 ± 0.4	92.0 ± 0.7	6.9 ± 0.0	3.7 ± 0.1	2.7 ± 0.6
T-120/AP		87.0 ± 0.5	3.3	45.6 ± 0.1	47.0 ± 0.0	8.3 ± 0.0	4.0 ± 0.0	40.8 ± 0.1
T-70	**16.8**	91.2 ± 0.8	2.8	51.6 ± 0.2	42.5 ± 0.0	7.5 ± 0.1	4.1 ± 0.1	46.0 ± 0.2
T-5	9.4	73.6 ± 0.7	2.7	30.9 ± 0.4	13.0 ± 0.1	10.8 ± 0.1	2.6 ± 0.3	73.7 ± 0.4
P-120	0.8	0.9 ± 0.3	4.4	46.3 ± 0.5	72.6 ± 0.1	9.4 ± 0.0	2.4 ± 0.0	15.7 ± 0.1
P-70/OP	2	19.8 ± 0.8	N.A.^d^	88.3 ± 0.6	79.1 ± 0.9	9.1 ± 0.1	1.7 ± 0.1	10.2 ± 0.9
P-70/AP		67.4 ± 0.6	3.5	135.2 ± 0.6	49.2 ± 0.0	7.3 ± 0.0	3.3 ± 0.1	40.2 ± 0.1
P-5	2.2	82.7 ±0.9	3.0	69.2 ± 0.4	43.7 ± 0.2	6.0 ± 0.5	3.0 ± 0.0	47.3 ± 0.7
P-0	**2.2**	0.5 ± 0.1	3.6	44.2 ± 0.2	58.8 ± 0.4	9.4 ± 0.2	2.0 ± 0.2	29.8 ± 0.4

a D = drying, T = torrefaction, P = pyrolysis; OP/AP = oily/aqueous phase; and number = condensation temperature.

b The amounts of raw materials were 6319, 6561, and 5950 g for aspen, goat willow, and rowan, respectively. The yields for the fractions having two phases were combined. The fraction with the highest yield is represented in boldface.

c Calculated on a dry weight basis; no sulfur was detected.

d N.A. = not analyzed due to high water content/small sample yield/physical form.

The overall acidity in all the fractions varied considerably. The pH values ranged between 3.2 and 4.5, and the TAN values were up to ∼100 mg/g, being the highest for the pyrolysis fractions, P-120 and P-70. These values are typical for wood-based fast and slow pyrolysis oils.^50^ While TAN is a direct measure of the total amount of acids present in pyrolysis oils, pH is dependent on the acid types (strong and weak) and the amount of water, being rather an indicator of the oil corrosiveness. The lowest TAN values were measured for the torrefaction fractions (ca. 1.5–4.5 mg/g), owing to the high content of water. The pH and TAN values were comparable to those in our previous work with birch bark,^10^ except that the pyrolysis fractions had much higher TAN values in the previous study, most likely due to the high content of suberinic fatty acids liberated from the birch bark. The elemental compositions did not vary considerably between the samples. In general, fractions with the lowest water content also had the lowest oxygen-to-carbon (O/C) ratios (on a dry weight basis), suggesting the presence of lipophilic extracts and other water-insoluble compounds. Some of the fractions had surprisingly high nitrogen content (up to ∼20 wt %). This is most likely due to the enhanced ability of short rotation trees of capturing inorganic nitrogen from the soil.^51^

### High-Resolution Mass Spectrometry

Molecular level compositions of the slow pyrolysis fractions were determined with high-resolution FT-ICR MS. To obtain a comprehensive view on the chemical compounds present in each fraction, two complementary ionization techniques, negative-ion ESI and positive-ion APPI, were used. While ESI is more sensitive toward polar, oxygen-containing compounds (e.g., acids, ketones, phenols, and carbohydrates), APPI allows detection of less polar compounds (e.g., neutral lipids, phenolics, and alicyclic or aromatic hydrocarbons). Only very small molecules (<50 Da) are not efficiently detected with FT-ICR MS, most of which are not very interesting from the utilization point of view of pyrolysis liquids. The benefit of high-resolution MS is that thousands of chemical substances can be directly detected without the need for compound derivatization.^43^

Up to 2000 compounds were detected in different liquid fractions with ESI/APPI FT-ICR MS. Overall, the number of compounds increased with the increasing process temperature. For the phase-separated fractions, a large amount of higher molecular weight compounds was detected in the oily as compared to the aqueous phases. The mass spectra obtained for the P-120 fractions were the most complex, having wide peak distributions up to *m*/*z* 600. In general, most of the peaks appeared around *m*/*z* 150–500 in the negative-ion ESI spectra. With positive-ion APPI, the drying and torrefaction fractions showed mass distributions from *m*/*z* 100 to 400, and the mass spectra had only some low-intensity peaks at *m*/*z* 200–300. There were obviously more peaks observed at a low mass range (*m*/*z* 100–200) as compared to ESI. Some fractions had very similar mass distributions as detected by both ESI and APPI.

Although high-resolution mass spectrometry provides unique elemental compositions for most detected analytes, allowing tentative compound identifications,^10^ this work concentrated in comparing the overall chemical compositions (i.e., lipids, sugars, phenolics, and hydrocarbons) between different liquid fractions. A vast majority of the compounds detected in each fraction belonged to the oxygen class (O_*x*_ class; *x* = 1–20 for ESI and 1–10 for APPI) with a minor contribution from sulfur- and nitrogen-containing compounds (S_*y*_O_*x*_ and N_*z*_O_*x*_ classes, respectively). In addition, hydrocarbons (HC class) were detected with APPI, and they were much more abundant in the pyrolysis fractions P-120, P-70, P-5, and P-0 and in the torrefaction fraction T-120. Therefore, only the O_*x*_ and HC classes were further analyzed in this work.

### Compounds Detected with Negative-Ion ESI

**Figure 1:**
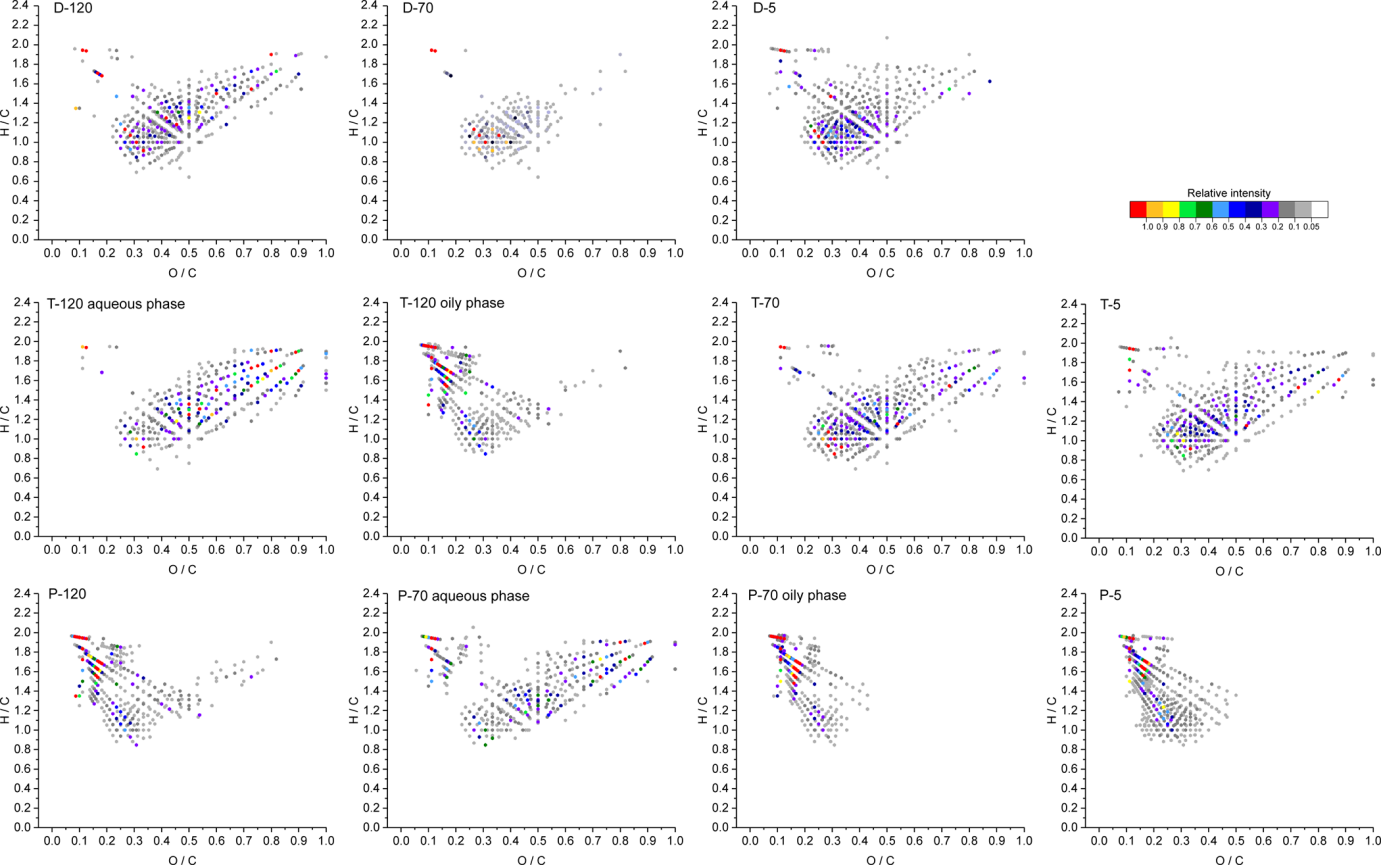
VK diagrams (color-coded for relative intensity) for aspen (*Populus tremula*) bark slow pyrolysis fractions based on negative-ion ESI FT-ICR MS data. The VK diagram for the fraction P-0 was almost identical to that of P-5 and is therefore not included.

The Van Krevelen (VK) diagram is a plot of the molar hydrogen-to-carbon (H/C) ratio as a function of oxygen-to-carbon (O/C) ratio for each detected compound with a distinct elemental composition and thus a powerful visualization tool for high-resolution mass spectral data of complex organic mixtures such as wood-based pyrolysis oils (see the example in Figure S2). The VK diagrams (color-coded for the relative intensity) for the compounds detected in the aspen bark pyrolysis fractions with negative-ion ESI are presented in Figure 1 (for the other tree species, see Figures S3 and S4). In addition, the double-bond equivalence (DBE) versus carbon number (C#) diagrams (combined for all O_*x*_ class compounds and hydrocarbons detected) are provided in Supporting Information Figures S5–S7.

Notable compositional differences were observed in the liquid fractions obtained at different process stages. When comparing the drying fractions D-120, D-70, and D-5, they all contained mostly condensed phenolic compounds (e.g., phenolic extractives). In addition, small amounts of lipids (mainly saturated fatty acids) and monosaccharides were observed as well (especially in the fractions D-120 and D-5). Otherwise, there were only very small differences between the different fractions obtained at the drying stage.

At the torrefaction stage, the liquid fractions for aspen and rowan, obtained at a condensation temperature of 120 °C (T-120), had two phases, while for goat willow, the liquid product remained in a single phase. The oily phase of T-120 was highly enriched with lipids (saturated and unsaturated fatty acids, fatty diacids, and other fatty acid derivatives), except for goat willow, for which T-120 contained mostly phenolic and sugaric compounds, similar to the other fractions of the torrefaction stage, T-70 and T-5. The high content of sugaric compounds (e.g., anhydrosugars and their derivatives) in the torrefaction fractions can be attributed to the preferential decomposition of hemicellulose at 275 °C.

The pyrolysis fractions P-120, P-70, P-5, and P-0 were highly enriched with lipids (e.g., suberinic fatty acids), lipophilic extractives, and triterpenes, except the aqueous phase of P-70, which instead contained mainly some phenolic compounds and sugars. In the cases of goat willow and rowan, the fractions P-5 and P-0 also contained some phenolic compounds.

**Figure 2:**
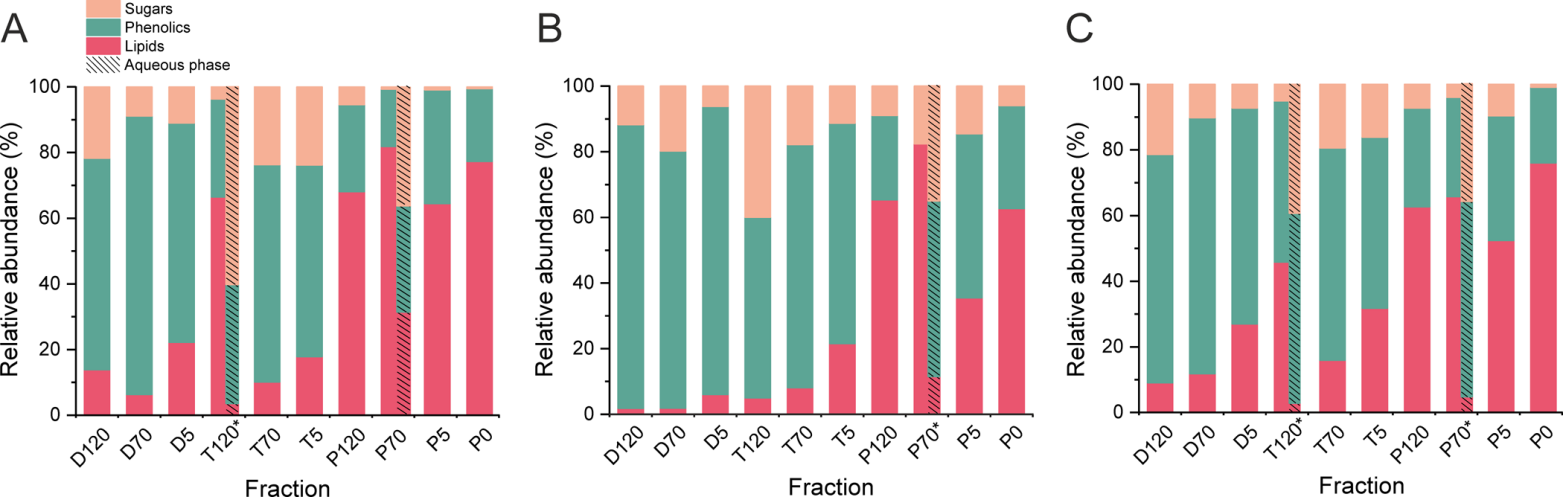
Relative proportions of lipids, phenolics, and sugars in the slow pyrolysis fractions of aspen (A), rowan (B), and goat willow (C) bark detected with ESI FT-ICR MS. The fractions marked with asterisk were phase-separated into oily and aqueous phases.

In order to compare the chemical compositions of each fraction more quantitatively, we defined different areas (H/C and O/C ratios) in the VK diagrams, representing different compound types (see Experimental Section for details), and calculated the sum intensities of all compounds in these areas. The relative proportions of the compound types detected in the slow pyrolysis liquid fractions of aspen bark with negative-ion ESI are presented in Figure 2.

As stated above, the number of lipids generally increased and the hydrophilic compounds (i.e., phenolics and sugars) decreased when the process temperature was raised. Most of these lipids belong to suberinic fatty acids and their derivatives. This result suggests that fatty acids are liberated from bark suberin only at relatively high temperatures (350 °C in our work), which is consistent with our earlier work with birch bark.^10^ This is also supported by the observation of phenolics, which are among the main building blocks of hardwood suberin. The highest amount of sugars was consistently produced in the torrefaction stage due to the efficient decomposition of hemicellulose.

### Compounds Detected with Positive-Ion Atmospheric Pressure Photoionization

**Figure 3:**
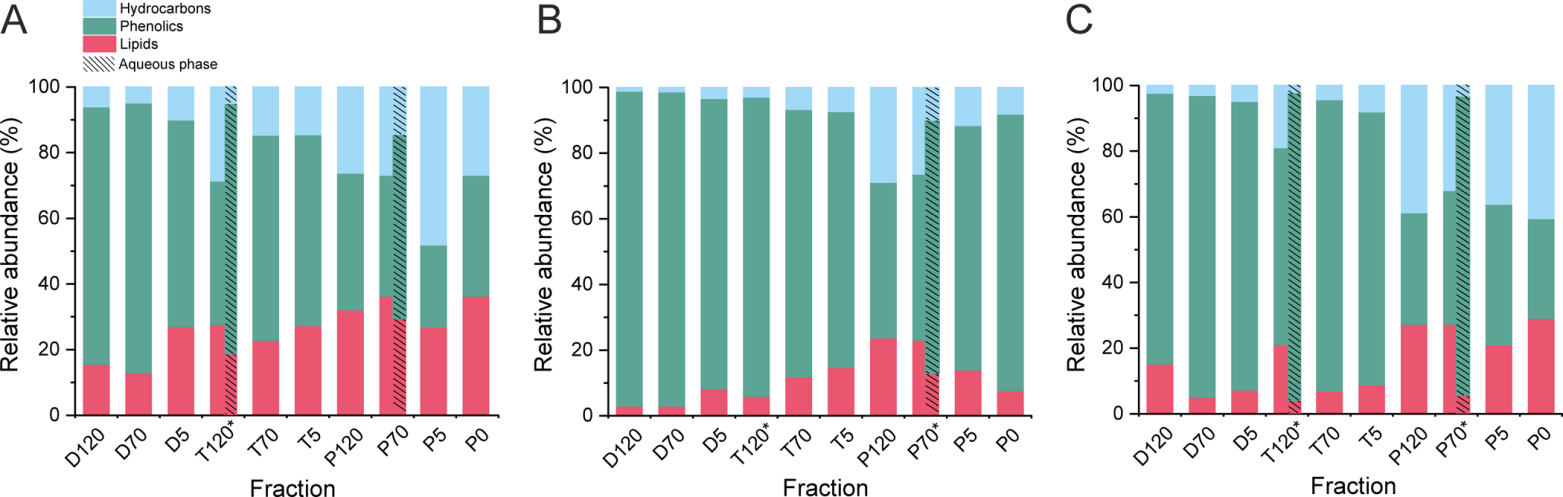
Relative proportions of lipids, phenolics, and hydrocarbons in slow pyrolysis fractions of aspen (A), goat willow (B), and rowan (C) bark detected with positive-ion APPI FT-ICR MS. The fractions marked with asterisk were phase-separated into oily and aqueous phases. For the breakout of different hydrocarbons, see Figure 4.

**Figure 4:**
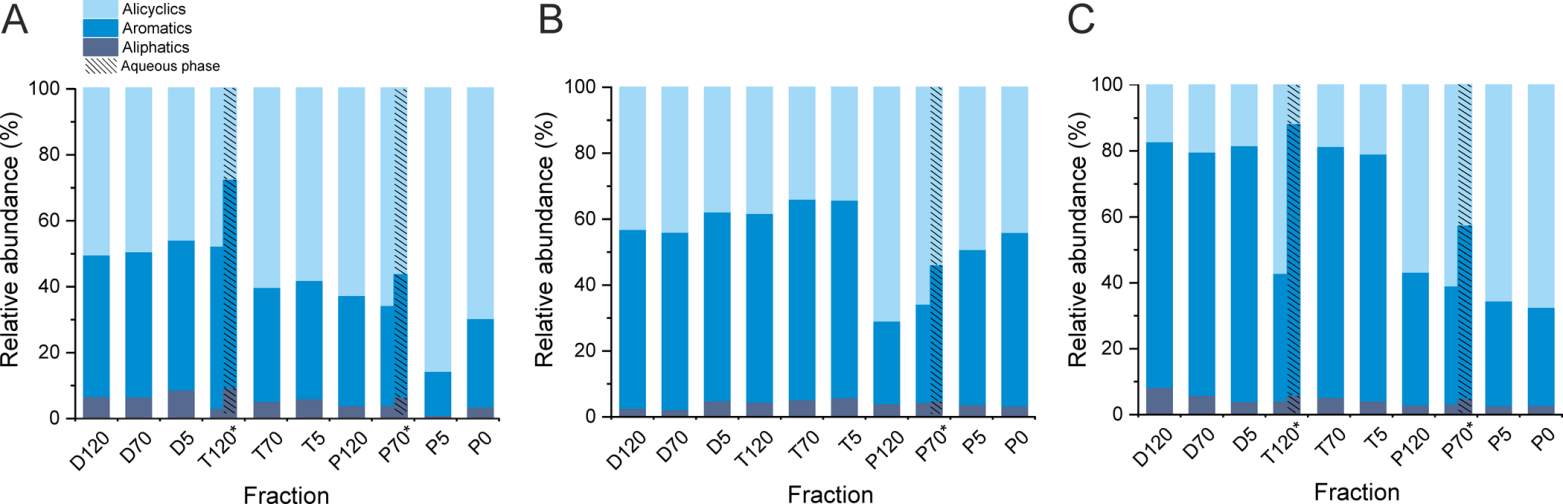
Relative proportions of different hydrocarbons (aliphatics, aromatics, and alicyclics) in slow pyrolysis fractions of aspen (A), goat willow (B), and rowan (C) bark detected with positive-ion APPI FT-ICR MS. The fractions marked with asterisk were phase-separated into oily and aqueous phases.

Since ESI cannot efficiently ionize less polar and non-polar pyrolysis liquid constituents (e.g., neutral lipids, some phenolic compounds and hydrocarbons), complementary measurements were performed with the positive-ion APPI technique, which is more sensitive toward these compound types. The VK diagrams (color-coded for relative intensity) for the compounds detected with positive-ion APPI are presented in Figures S8–S10 and the relative amounts of the compounds are presented in Figure 3. In addition, Figure 4 shows the relative proportions of different hydrocarbons in the fractions. Since all hydrocarbons reside at O/C = 0 in a conventional VK diagram, the DBE versus C# plots (Figures S11–S13) were used to differentiate between aliphatic, alicyclic, and aromatic hydrocarbons. In pyrolysis liquids, a majority of HC class compounds are derived from lignin degradation, producing mainly aromatic (or polyaromatic, PAH) hydrocarbons, and dehydration of alicyclic compounds (e.g., triterpenoids), resulting primarily in the formation of alicyclic HCs, and eventually PAHs at the highest pyrolysis temperatures. In practice, paraffinic hydrocarbons (alkanes) do not usually form in conventional pyrolysis conditions, but minor amounts of olefinic hydrocarbons may form due to fatty acid dehydration and decarboxylation.

The total amount of hydrocarbons increased with the increasing pyrolysis temperature, as expected. In contrast, the amount of phenolics decreased being the lowest for the pyrolysis fractions. Based on the DBE versus C# plots (Figures S10–S13), APPI preferentially ionized monophenolics, whereas ESI ionized larger and more polar phenolic constituents, including phenolic extractives. Thus, ESI and APPI provide complementary compositional information for these types of compounds. No major differences were observed between different tree species, except that goat willow had slightly higher content of lipids in all the liquid fractions. Alicyclic HCs were primarily resulting from dehydration/dealkylation of triterpenoids based on the DBE and carbon number distributions (DBE = 5–10, C# = 25–30). Aromatic HCs comprised mainly alkylbenzenes and the like (DBE = 4–8, C# = 5–15). Aliphatic compounds consisted of (fatty) acids and acid esters with DBE ≤ 4. The liquid fractions obtained from the pyrolysis stage had more alicyclic and less aromatic HCs than those from the drying and torrefaction stages. The oily phase had a higher number of alicyclic hydrocarbons than the aqueous phase, which was consistent with the mass distributions observed in the APPI MS spectra. Interestingly, the drying and torrefaction fractions of rowan bark had a higher content of aromatic hydrocarbons (up to 80%) than the corresponding fractions of aspen and goat willow.

## Conclusions

The temperature-programmed slow pyrolysis was used for thermochemical conversion of bark obtained from three short rotation tree species, namely, aspen, goat willow, and rowan. The fractional condensation applied at three different temperatures (except four in the pyrolysis stage) resulted in 10 distinct liquid fractions whose chemical compositions varied considerably. The heating temperature had the biggest influence on the chemical compositions of the liquid fractions. The drying fractions comprised mainly small phenolic extractives and water. Also small amounts of lipids and sugars were detected. Thus, these fractions possess no economic value. The torrefaction fractions comprised mainly sugars and phenolic compounds. For these fractions, the water content increased with the decreasing condensation temperature, except for goat will for which the water content was 93–99 wt % in all torrefaction fractions. The phase separation was observed in the T-120 fractions of aspen and rowan due to much higher content of lipophilic extractives as compared to T-70 and T-5. These compounds were highly enriched in the oily phases. In contrast, the pyrolysis fractions had much higher content of lipids (e.g., suberinic fatty acids, triterpenes, and their derivatives) than the other fractions. Moreover, the pyrolysis fractions had a higher content of alicyclic and aromatic hydrocarbons, resulting from the extensive dehydration/decarboxylation reactions occurring at higher temperatures. Relative proportions of different hydrocarbons varied considerably between different tree species. The present results suggest that the temperature-programmed slow pyrolysis with fractional condensation offers a potential valorization route for tree bark residues, resulting in liquid fractions with distinct chemical compositions. These fractions could be used in different applications or upgraded/fractionated further.

## Experimental Section

### Feedstocks

Bark samples were collected from three different short rotation tree species (i.e., aspen, goat willow, and rowan) in March 2018 during the winter dormancy of the trees. The naturally regenerated trees were grown in the research forest of Natural Resources Institute of Finland (LUKE), located at Punkaharju, Finland (61° 80′ N, 29° 32′ E). The trees (one stem of goat willow and aspen and two stems of rowan) were felled and cut, and all the bark layers from surface to xylem were peeled off from the lower parts of the frozen stems. The bark samples were kept frozen until processed using slow pyrolysis. The dimensions of the trees harvested for bark sampling and the total amount of bark obtained for each tree species are given in Table 2.

**Table 2:** Dimensions of the Trees Harvested for Bark Sampling

species	diameter at stump height^a^ (cm)	bark thickness (cm)	total amount of bark obtained (g)
Aspen	27.1	0.7	6319
Goat willow	15.8	0.4	6561
Rowan^b^	14.0	0.3	5950

a Bark included.

b Mean values of two stems.

### Slow Pyrolysis Experiments

The frozen fresh barks were first compressed at a pressure of 200 bar to remove the residual free water. The pyrolysis process and the reactor setup have been described in detail earlier (for brief technical description, see the Supporting Information).^52^ A schematic diagram of the pyrolysis reactor is shown in Figure S1. Briefly, heating was performed in three stages, drying, torrefaction, and slow pyrolysis, occurring at temperatures of 135, 275, and 350 °C, respectively. In each stage, three nominal condensation temperatures of 120, 70, and 5 °C were applied (in the slow pyrolysis stage, an additional fraction was also collected at 0 °C). The approximate residence times were 18–22 h in the drying stage, 19–21 h in the torrefaction stage, and 6–8 h in the slow pyrolysis stage. At the end, 10 liquid fractions were obtained for each tree species.

### Bulk Chemical Analyses

Bulk chemical analyses consisted of TAN, pH, water content, and elemental composition analyses. The SI Analytics Titronic universal titrator (SI Analytics GmbH, Mainz, Germany) was used to determine the TAN values by following the ASTM D3339 standard, and the pH values were measured using a PHM210 standard pH meter (Radiometer Analytical, Villeurbanne Cedex, France) equipped with a Red Rod electrode. The water content was determined by Karl Fisher titration on a Metrohm 870 KF Titrino Plus titrator (Metrohm AG, Herisau, Switzerland) using a volumetric ASTM E203-08 method. Vario Micro Cube V1.7 apparatus (Elementar Analysensysteme GmbH, Hanau, Germany) was used to determine the elemental (CHNOS) compositions of the samples, using sulfanilamide (C_6_H_8_N_2_O_2_S) as a reference. The amount of oxygen was calculated by difference (O % = 100% – CHNS %). The results from the bulk chemical analyses have been reported as average ± 1 standard deviation over several replicate samples.

### High-Resolution Mass Spectrometry

To prepare stock solutions for mass spectrometry, each sample was accurately weighed and diluted with methanol to a concentration of 1 mg/mL (on a dry weight basis). The solubility was then visually inspected. The prepared stock solutions were further diluted with methanol (for ESI) or a mixture of methanol and toluene (1:1, v/v) (for APPI) to a final concentration of 200 μg/mL and measured directly. All solvents used in the MS analyses were of HPLC grade.

All the samples were analyzed on a 12-T Bruker Solarix XR FT-ICR MS (Bruker Daltonics, Bremen, Germany), equipped with an Apollo-II atmospheric pressure ion source, a dynamically harmonized ICR cell (ParaCell), and an actively shielded 12 T superconducting magnet. Negative-ion ESI and positive-ion APPI techniques were applied. The details of data acquisition and post-processing can be found elsewhere.^10^

For the molecular formula assignment, the parameters were as follows: elemental composition, ^12^C_0–50_^1^H_0–150_^14^N_0–2_^16^O_0–30_^32^S_0–1_; mass error ≤ 0.5 ppm; DBE = 0–25; signal-to-noise (S/N) ≥4; H/C ratio ≤3; and mSigma ≤ 1000. The final MS data were sorted in Microsoft Excel 2016 and further visualized by using Origin Pro 9.1 software. The compounds were grouped into different compound classes (e.g., O_*x*_ = the compounds containing a variable amount of carbon and hydrogen atoms and *x* oxygen atoms) and their sum relative intensities were computed. The DBE values were calculated from the equation DBE = *c* – 1/2*h* + *n* + 1, for any compound having a general formula of C_*c*_H_*h*_N_*n*_O_*o*_S_*s*_.

The VK diagrams were used as a main visual means for the comparison of liquid fraction compositions.^53^ In addition, different ranges of H/C and O/C ratios were defined for different classes of oxygen-containing compounds (i.e., lipids, sugars, and phenolics), and their sum intensities were calculated. Although this kind of categorization is not perfect as the H/C and O/C ratios are not well defined for different compounds, it gives a sufficient overview on the relative compound distributions in each sample. Hydrocarbons, detected only with APPI, were further classified into three distinct compound classes (aliphatic, alicyclic, and aromatic hydrocarbons) based on their DBE and carbon number (C#) values. Table S1 summarizes the H/C and O/C (oxygenates) or DBE and C# values (hydrocarbons) used for the compound categorization. The values (slopes and intercepts) were chosen so that none of the compounds could be within the line and therefore not counted. An exemplary VK diagram for aspen bark torrefaction fraction (T-5) is shown in Figure S12. The final sum intensities were calculated in Microsoft Excel by using a COUNTIFS function.

## Supporting Information Available

Technical description of the pyrolysis reactor setup; schematic diagram of the slow pyrolysis reactor, exemplary van Krevelen diagrams, and DBE versus carbon number plots; and values of H/C, O/C, DBE, and C#. The raw mass spectrometry data is available from the authors upon reasonable written request (PDF)
