# Supplementary material for: Valorization of Bark from Short Rotation Trees by Temperature-Programmed Slow Pyrolysis
Source: ACS Omega. 2021 Mar 31;6(14):9771–9. doi: 10.1021/acsomega.1c00434 (PMC8047738; doi:10.1021/acsomega.1c00434)
Supplement: Supplementary file 1 — ao1c00434_si_001.pdf [file ao1c00434_si_001.pdf]

Supporting Information

Valorization of Bark from Short Rotation Trees by Temperature-Programmed Slow Pyrolysis

Qing Zhao[a,b], Marko Mäkinen[a], Antti Haapala[b], Janne Jänis[a]*

[a]Department of Chemistry, University of Eastern Finland, FI-80100 Joensuu, Finland

[b]School of Forest Sciences, University of Eastern Finland, FI-80100 Joensuu, Finland

*Corresponding author: janne.janis@uef.fi

This file contains:

Technical description of the slow pyrolysis reactor (page S-2)

Supporting Table S1 (page S-3)

Supporting Figures S1–S13 (Pages S-2, S-4 to S-14)

Number of pages: 13

Number of Tables: 1

Number of figures: 13

Technical description of the slow pyrolysis reactor

The slow pyrolysis reactor consists of a ca. 10-L (30 cm diameter) steel chamber, equipped with an electrical heater (**Figure S1**). The typical heating rate was 2 ℃/min. During the heating, a carrier gas of CO_2 was fed into the reactor at a constant flow rate of 2 L/min. The heating was performed in three stages: the drying stage (22–135 ℃; hold time at 135 ℃ of 18-22 h), torrefaction stage (135–275 ℃; hold time at 275 ℃ of 19-21 h) and pyrolysis stage (275–350 ℃; hold time at 350 ℃ of 6-8 h). The pyrolysis liquids (distillates) were obtained from three separate condenser units, operated at 120, 70, and 5 ℃. In this work, an additional condenser operated at 0 ℃ was used. The condenser temperatures were controlled by VWR model 1157 P (VWR International, Vienna, Austria) and WK1200 Lauda (Lauda-Brinkmann, Delran, NJ, USA) circulation chillers. The reactor system was controlled and monitored by Hotwell (Hotwell Oy, Mikkeli, Finland) and Siemens Logo (Siemens AG, Munich, Germany) software.

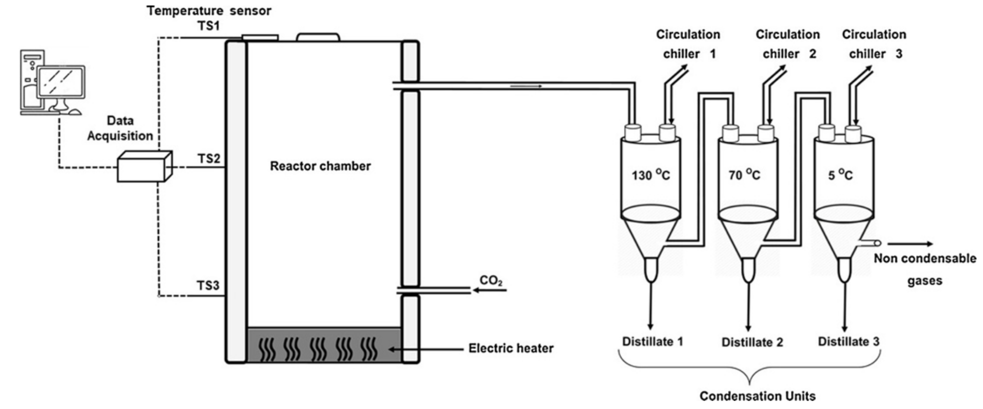

Figure S1. Schematic diagram of the slow pyrolysis reactor used in this study (Figure adapted from: Salami A. *et al.*, *Industrial Crops & Products* **2020**, 155: 112760)

Table S1. Values of H/C, O/C, DBE, and C# used for categorization of different compounds.

Compound class	Detected with (−)ESI	Detected with (+)APPI	
	H/C, O/C	H/C, O/C	DBE, C#
**Lipids**	$H/C > 2.72 \times O/C + 0.71$<br>$H/C < -2.99 \times O/C - 2.99$	$H/C > 5.38 \times O/C + 0.93$<br>$O/C > 0$	
**Sugars**	$H/C > -2.99 \times O/C - 2.99$	N.D.	
**Phenolics**	$H/C < 2.72 \times O/C + 0.71$<br>$H/C < -2.99 \times O/C - 2.99$	$H/C < 5.38 \times O/C + 0.93$<br>$O/C > 0$	
**Hydrocarbons**	N.D.	$O/C = 0, H/C = 0–3$	
Aliphatics			$DBE < 4$
Alicyclics			$DBE \geq 4, DBE < 0.69 \times C\# - 6.23$
Aromatics			$DBE \geq 4, DBE > 0.69 \times C\# - 6.23$

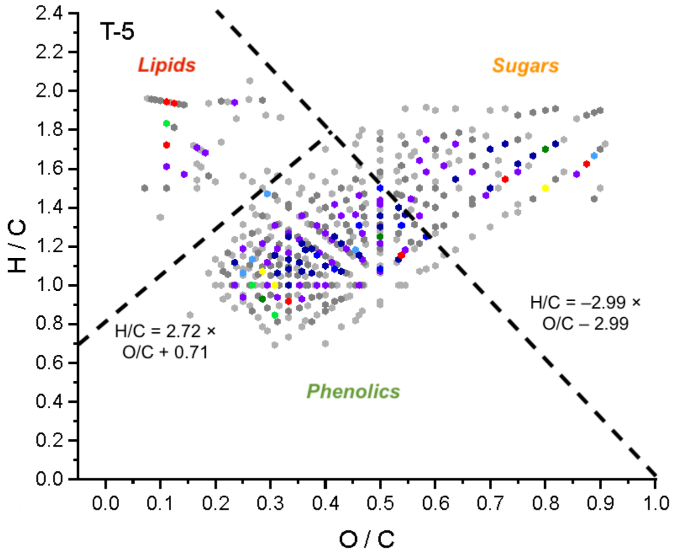

Figure S2. Exemplary van Krevelen diagram for negative-ion ESI FT-ICR MS data of aspen bark torrefaction fraction T-5), showing boundaries of H/C and O/C values used for compound class categorization (see, Table S1 for details).

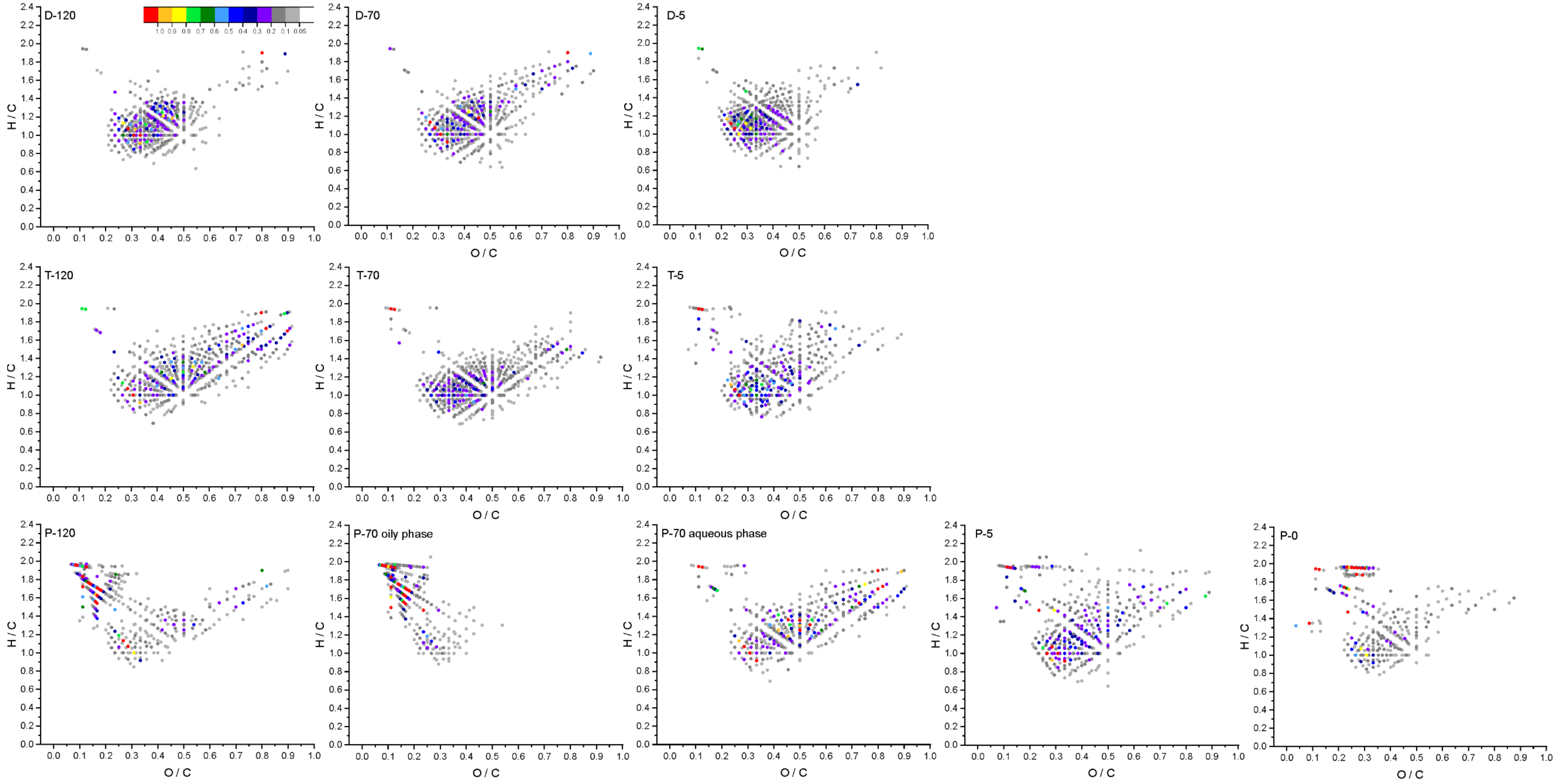

Figure S3. Van Krevelen diagrams (color-coded for relative intensity) of goat willow bark slow pyrolysis liquids based on negative-ion ESI.

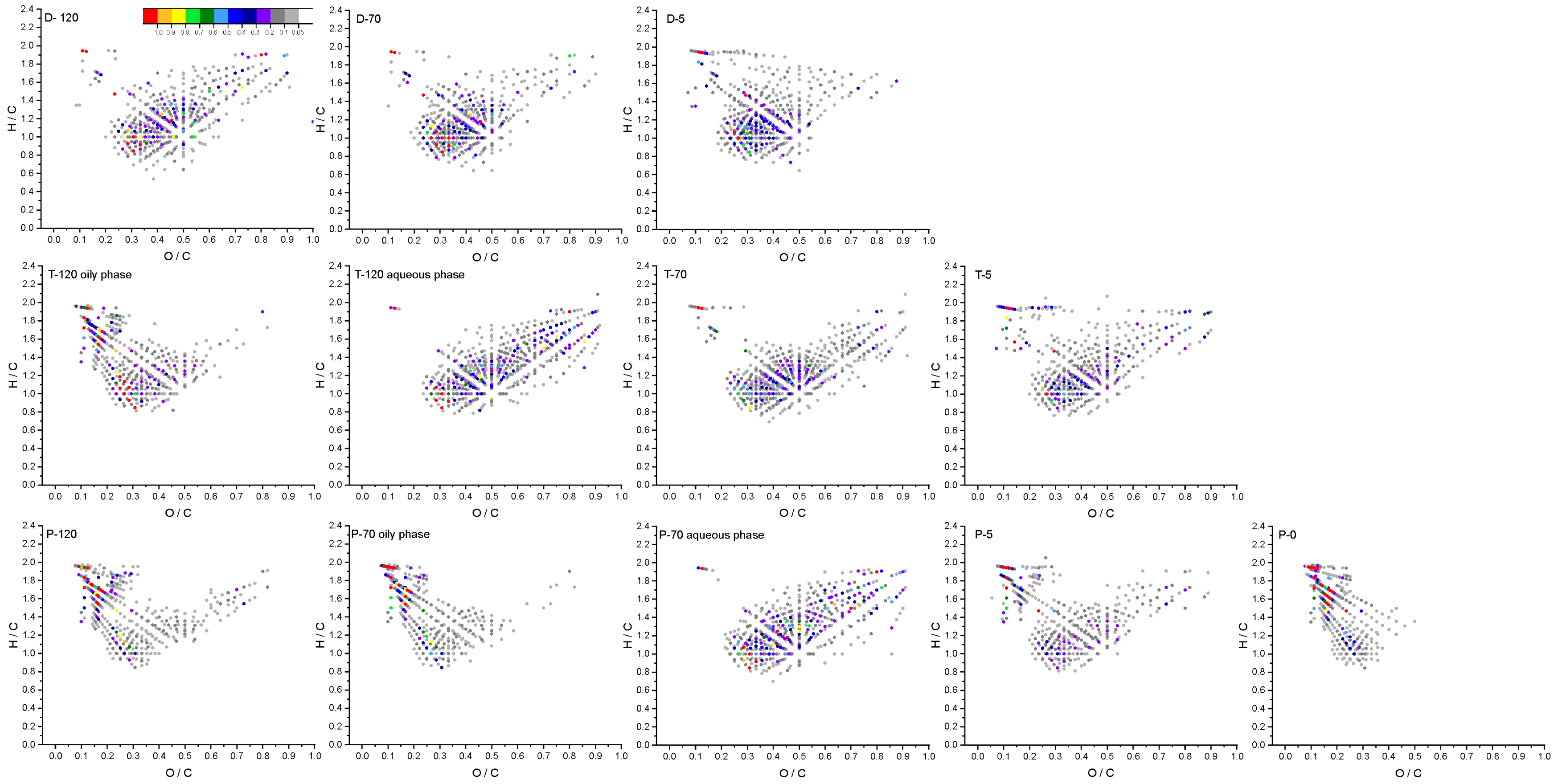

Figure S4. Van Krevelen diagrams (color-coded for relative intensity) of rowan bark slow pyrolysis liquids based on negative-ion ESI.

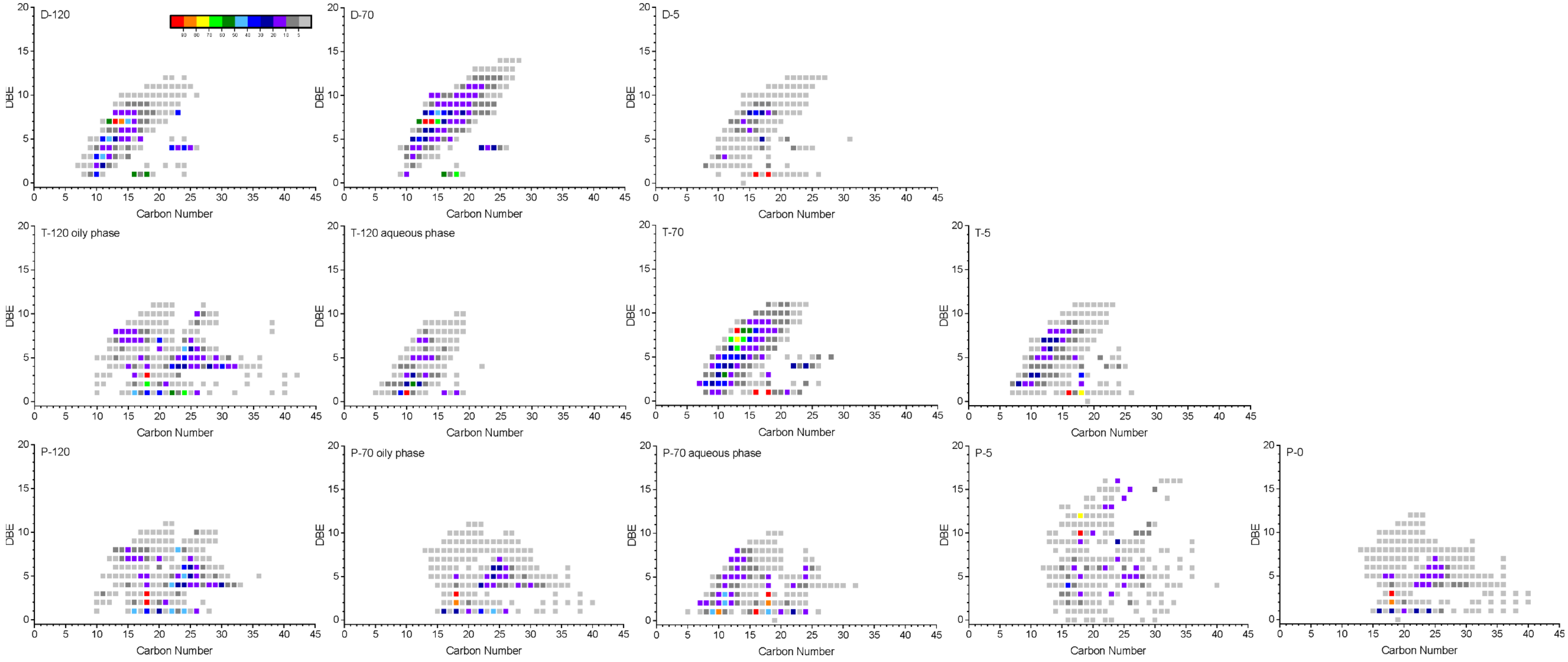

Figure S5. Combined double bond equivalent (DBE) vs. carbon number plots of all oxygen classes and hydrocarbons detected by negative-ion ESI in aspen bark slow pyrolysis liquids.

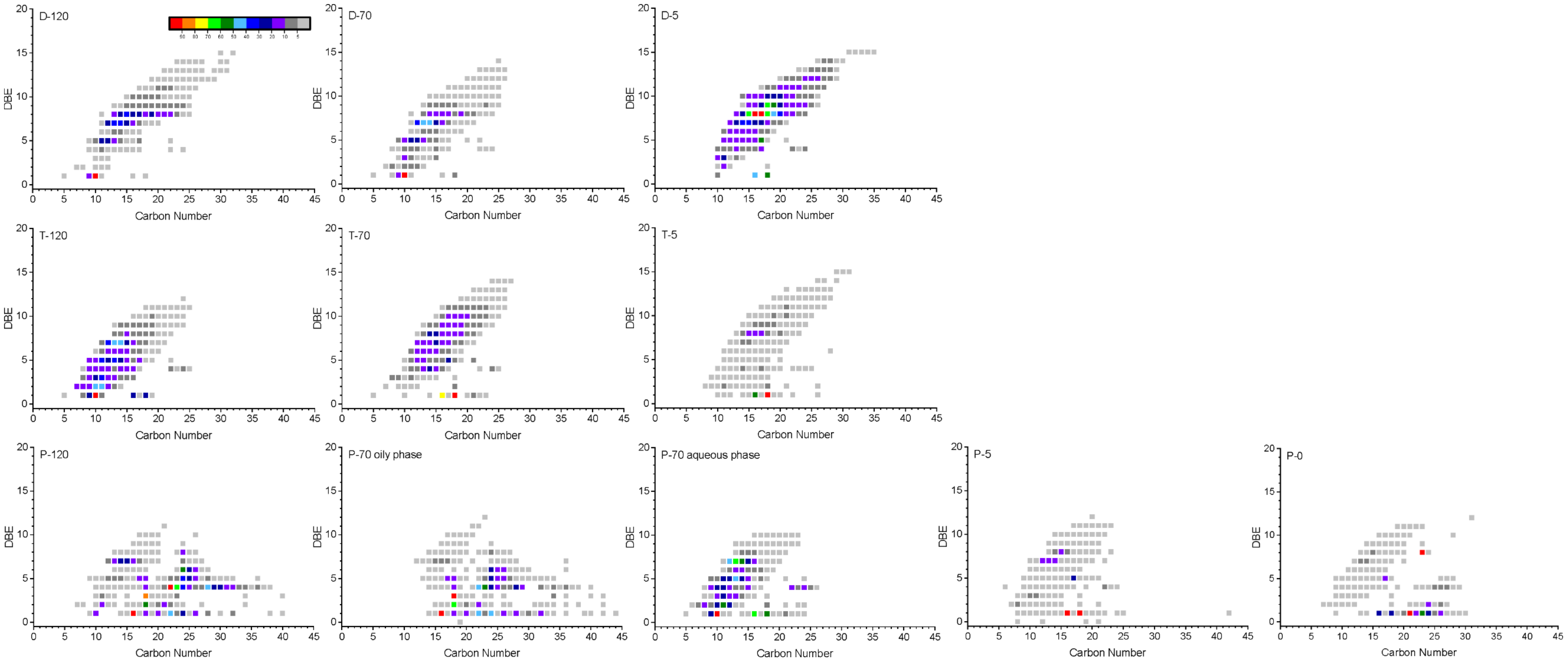

Figure S6. Combined double bond equivalent (DBE) vs. carbon number plots of all oxygen classes and hydrocarbons detected by negative-ion ESI in goat willow bark slow pyrolysis liquids.

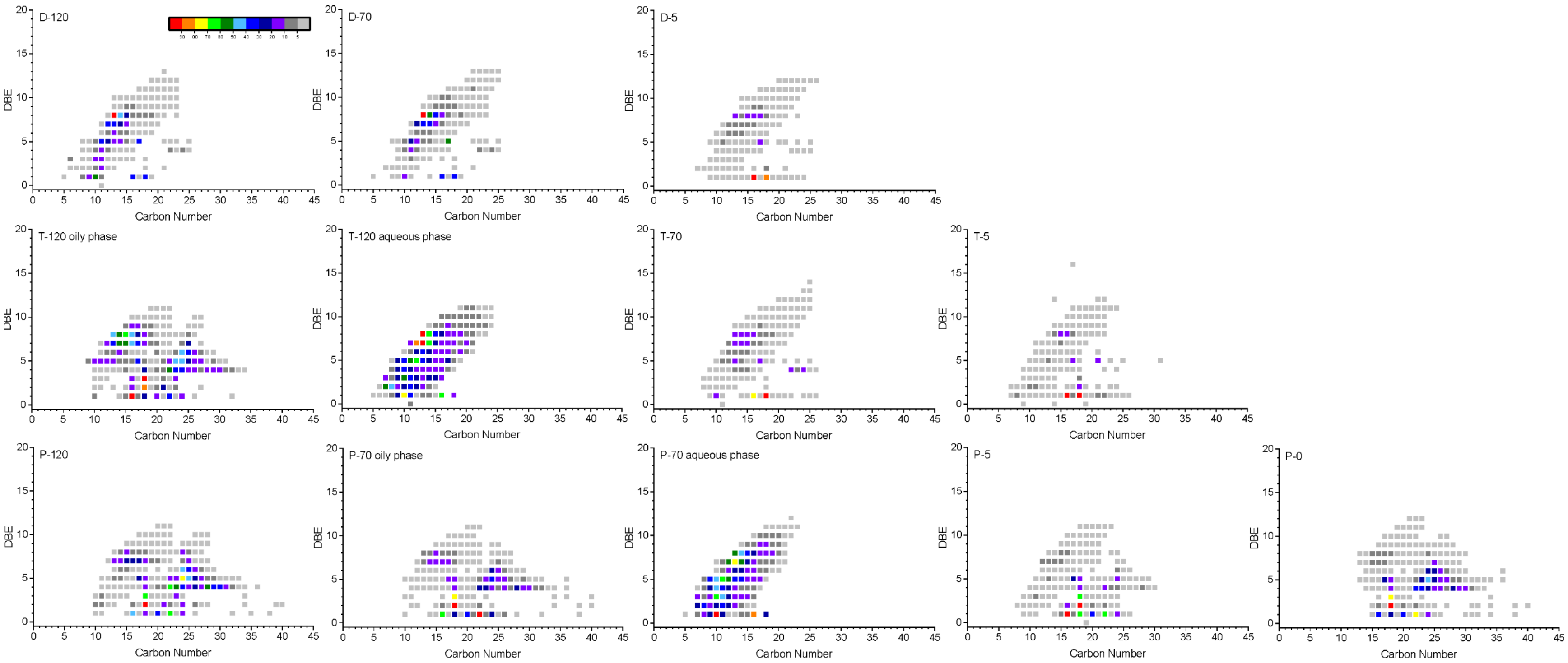

Figure S7. Combined double bond equivalent (DBE) vs. carbon number plots of all oxygen classes and hydrocarbons detected by negative-ion ESI from rowan bark slow pyrolysis liquids.

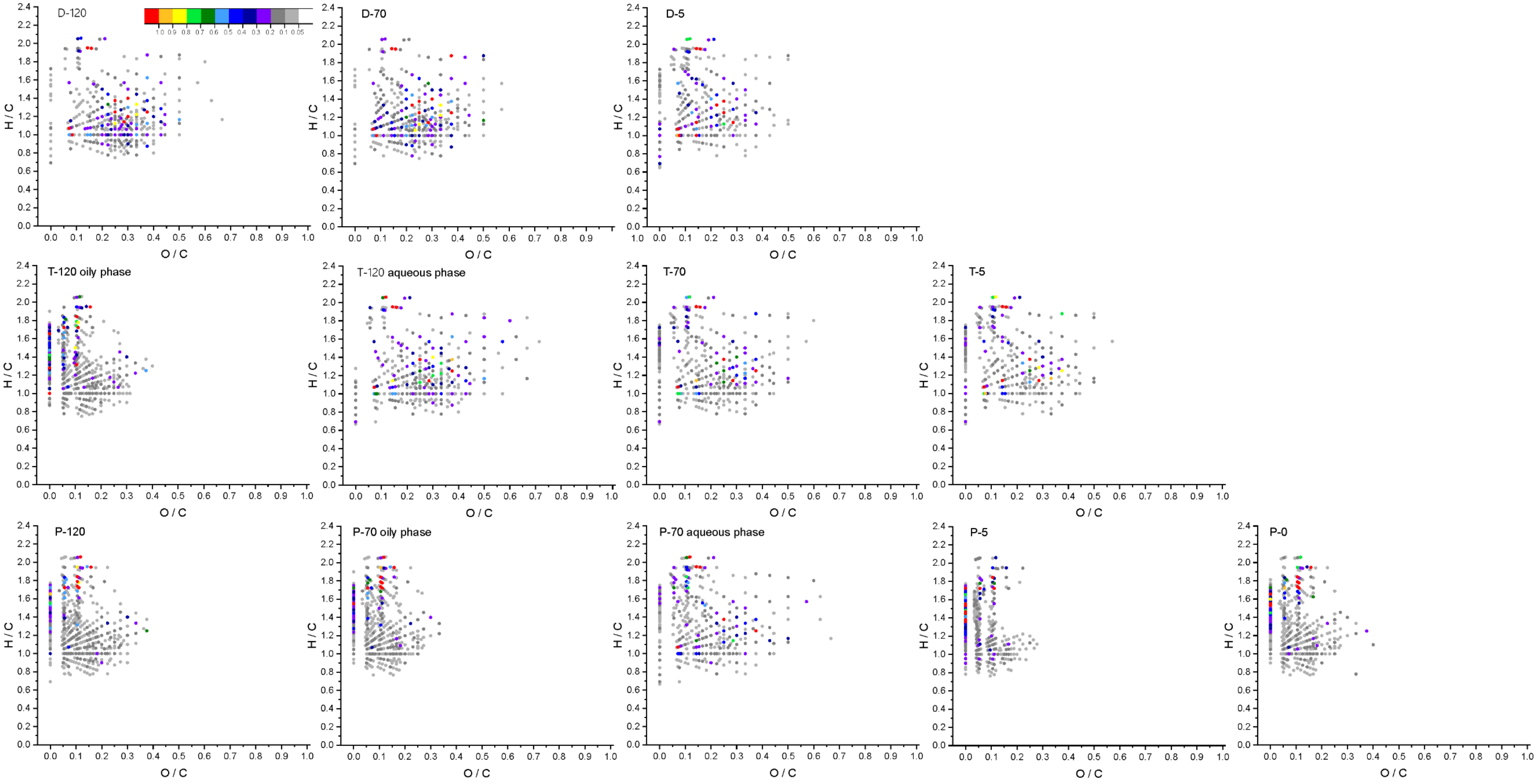

Figure S8. Van Krevelen diagrams (color-coded for relative intensity) of aspen bark slow pyrolysis liquids identified with positive-ion APPI.

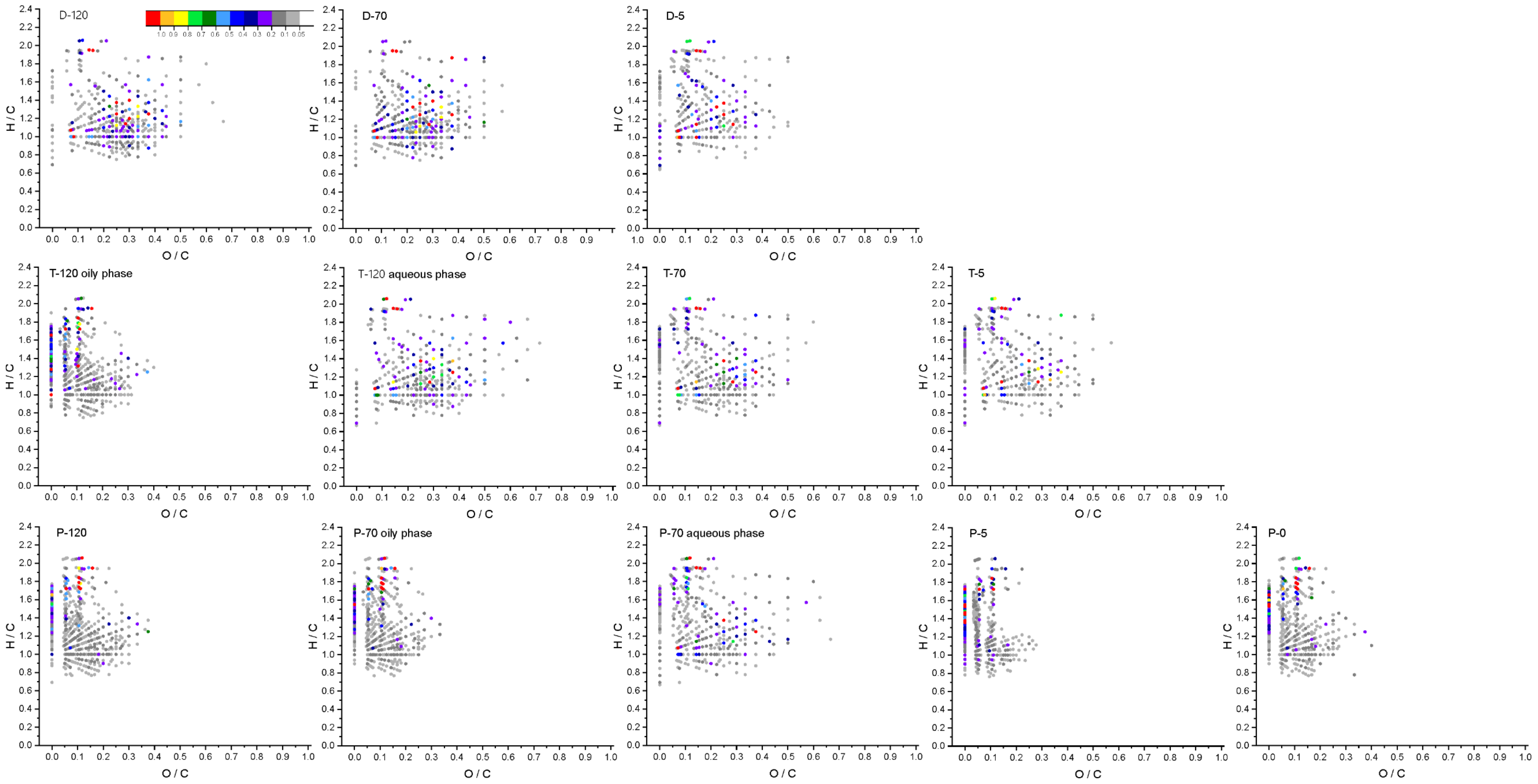

Figure S9. Van Krevelen diagrams (color-coded for relative intensity) of goat willow bark slow pyrolysis liquids identified with positive-ion APPI.

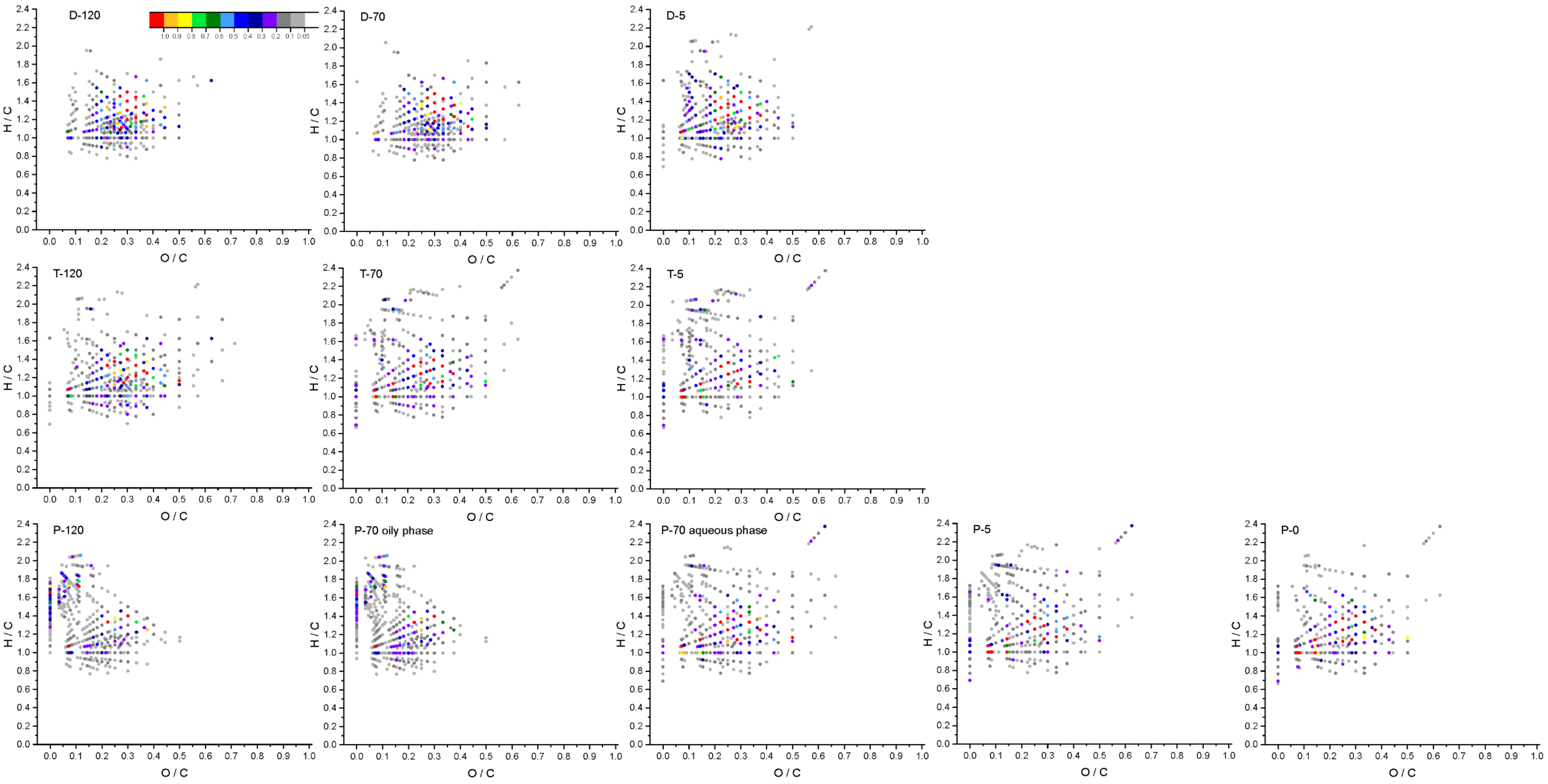

Figure S10. Van Krevelen diagrams (color-coded for relative intensity) of rowan bark slow pyrolysis liquids identified with positive-ion APPI.

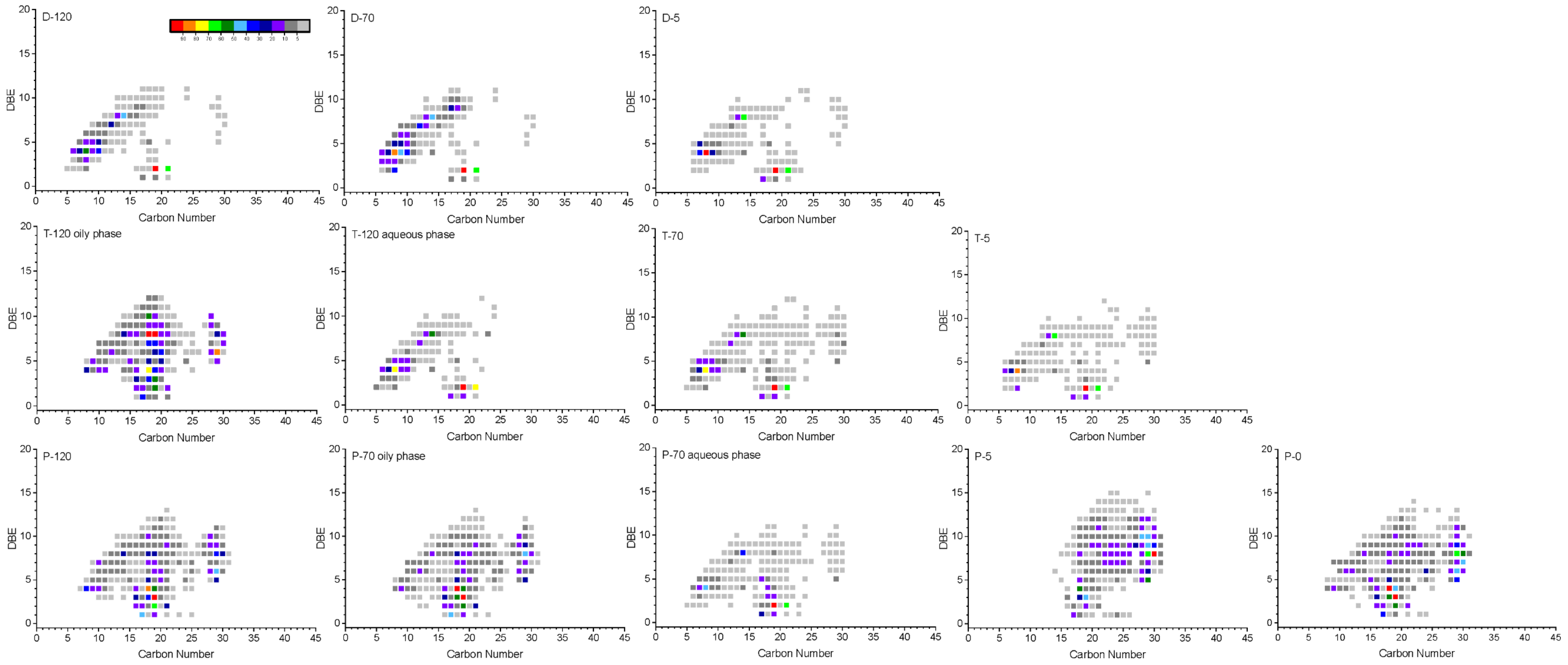

Figure S11. Combined double bond equivalent (DBE) vs. carbon number plots of all oxygen classes and hydrocarbons detected by positive-ion APPI from aspen bark slow pyrolysis liquids.

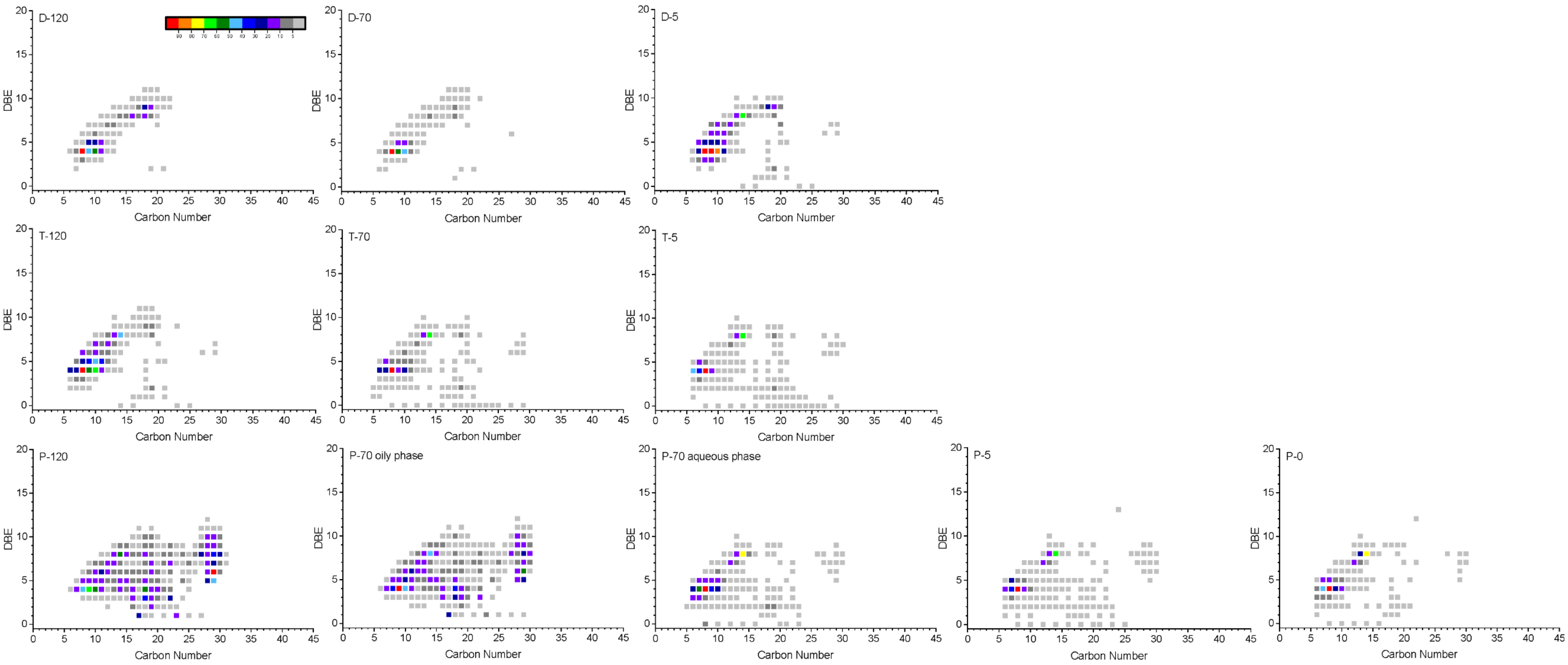

Figure S12. Combined double bond equivalent (DBE) vs. carbon number plots of all oxygen classes and hydrocarbons detected by positive-ion APPI from goat willow bark slow pyrolysis liquids.

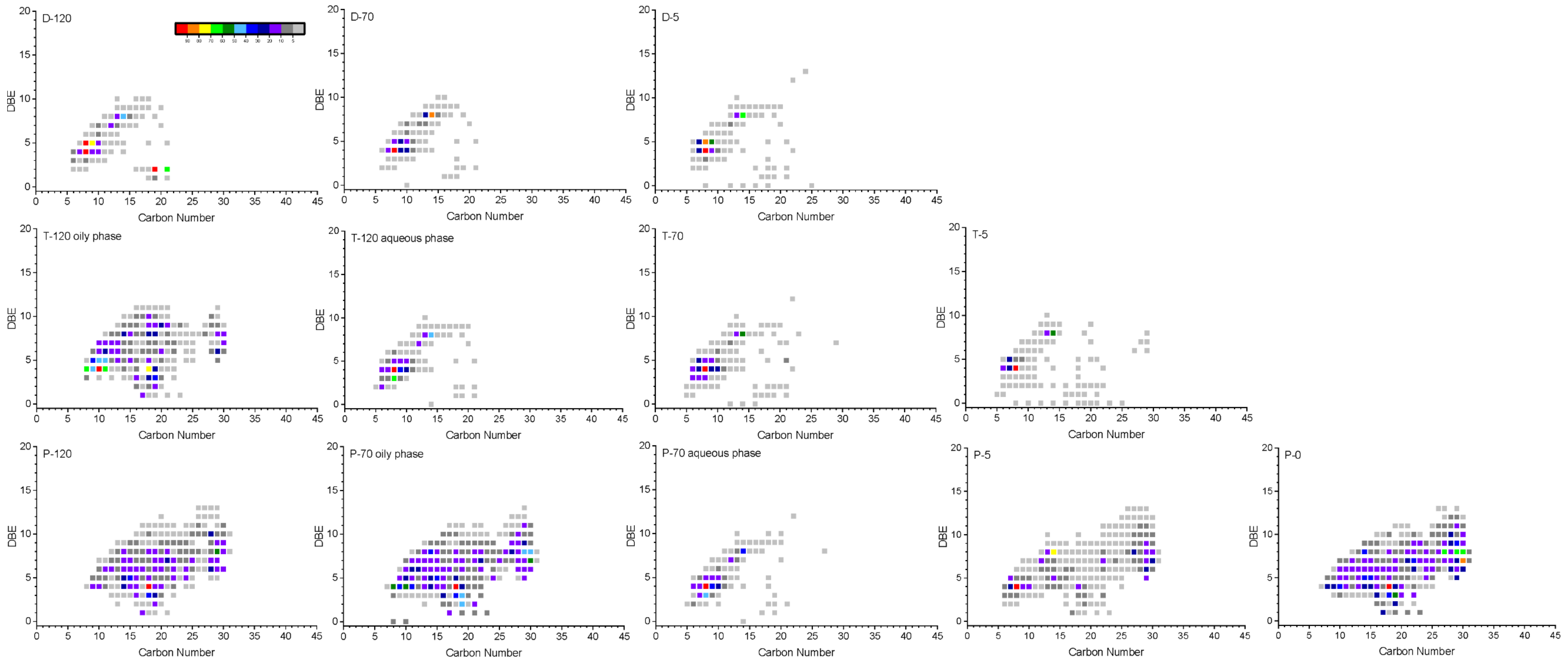

Figure S13. Combined double bond equivalent (DBE) vs. carbon number plots of all oxygen classes and hydrocarbons detected by positive-ion APPI from rowan bark slow pyrolysis liquids.
